# Improvements to Load-Bearing Capacity and Settlement of Clay Soil After Adding Nano-MgO and Fibers

**DOI:** 10.3390/polym17141895

**Published:** 2025-07-09

**Authors:** Baki Bağrıaçık, Barış Mahmutluoğlu, Szymon Topoliński

**Affiliations:** 1 Faculty of Engineering, Civil Engineering Department, Cukurova University, Balcali 01330, Adana, Turkey; bakibagriacik@gmail.com; 2 Vocational School of Technical Sciences, Construction Department, Mersin University, Mersin 33110, Mersin, Turkey; barismahmutluuoglu@gmail.com; 3 Department of Road Engineering, Transport and Geotechnics, Faculty of Civil and Environmental Engineering and Architecture, Bydgoszcz University of Science and Technology, 85-796 Bydgoszcz, Poland

**Keywords:** soil improvement, nano, fibers, bearing capacity, settlements, model test

## Abstract

Recently, the utilization of nanomaterials has been gaining popularity in soil improvement procedures. The aim of this study is to investigate the feasibility of using nano-MgO (NM) along with fibers (FBRs) to improve clay soil’s load-bearing capacity and settlement by using both conventional and model experiments. First, the optimum water content values for NM–clay mixtures were determined by compaction tests. For mixtures prepared with the optimum water content, the addition of 1.5% NM alone resulted in a 1.33-times improvement in bearing capacity, and the addition of 1.5% 90 mm FBRs alone led to a 1.83-times improvement. Utilizing 1.0% NM and 1.5% FBRs at 90 mm long resulted in a 3.07-times improvement in bearing capacity. The temperature of the mixing water was found not to be a significant parameter, and curing duration impacted the results only for NM addition. Significant settlement improvements were achieved by adding the two materials together. Therefore, utilizing NM and FBRs together resulted in an optimal outcome for clay soil.

## 1. Introduction

The rapid expansion of urbanization and infrastructure development has led to an increasing scarcity of land that meets the required geotechnical and economic conditions for safe construction. Areas with suitable soil properties are becoming increasingly limited and often prohibitively expensive. Consequently, ground improvement techniques such as chemical stabilization, grouting, and compaction have become essential for enabling the use of problematic or marginal soils. However, these traditional methods are typically associated with high implementation costs, intensive labor requirements, and time-consuming procedures, thereby limiting their large-scale applicability [1]. In response to these challenges, numerous studies have explored the use of alternative soil additives derived from industrial and agricultural waste. Materials such as end-of-life tires [2], pond ash [3], cement kiln or bypass dust [4], construction and demolition debris [5], rice husk ash [6], glass manufacturing waste [7], waste stone powder [8], geopolymers [9], biopolymers [7], steel slag [10], fly ash [11], and recycled asphalt pavement [12] have been investigated for their potential to enhance soil performance. While many of these materials have shown promise, their environmental impact and variability in performance remain concerns. Traditional chemical stabilizers, although effective, often pose serious environmental risks. They can release harmful gases, including CO_2_, SO_2_, and NO_X_, and may introduce toxic by-products into soil and groundwater following application [13]. These environmental concerns have led to an increased interest in sustainable and environmentally friendly alternatives that maintain or improve soil performance while reducing ecological impact. In this context, nanomaterials have emerged as a new generation of soil improvement agents. Their high specific surface area, nanoscale particle size, and enhanced physicochemical reactivity allow them to effectively interact with fine-grained soils, leading to significant improvements in engineering properties even at very low concentrations [14]. However, despite their considerable potential, the application of nanomaterials in geotechnical engineering remains relatively limited compared to other civil engineering fields. Moreover, ecologists have emphasized the need for systematic ecological risk assessments due to the uncertain long-term environmental effects of nanomaterials [15]. Several nanomaterials, including nano-bentonite, nano-laponite, nano-alumina, nano-silica, nano-clay, nano-copper, nano-CuO, nano-MgO, carbon nanotubes, and nano-zeolite, have demonstrated promising performance in soil treatment applications [16]. Among these, nano-silica is particularly well studied. Research has shown that even minimal additions (0.7–3%) of nano-silica, either independently or in combination with conventional binders such as cement or lime, can significantly enhance soil compressibility resistance, unconfined compressive strength, and microstructural cohesion [17,18,19,20,21,22,23,24,25,26,27,28]. Carbon nanotubes (CNTs), composed of rolled graphene sheets, offer exceptional mechanical properties, including tensile strengths up to 100 times greater than steel and six times lower density [29]. Their incorporation into soil at small concentrations (1–3%) has been found to substantially improve compressive strength and stiffness [30]. Similarly, nanomaterials such as nano-bentonite [31,32] and nano-laponite [33,34] have demonstrated remarkable effectiveness in improving soil resistance to liquefaction and increasing compressive strength. Nano-clay, particularly nano-montmorillonite, has also been shown to significantly enhance unconfined compressive strength and California Bearing Ratio (CBR) values at low dosages, such as 1.5% [35]. Despite these promising outcomes, the literature clearly reveals that the application of nanomaterials in geotechnical engineering is still in its early stages.

Comprehensive experimental studies, particularly large-scale and field-oriented investigations, remain limited. This indicates a notable gap in knowledge and a need for further exploration of nanomaterials’ mechanisms and effectiveness under diverse geotechnical conditions. This study aims to address this gap by investigating the combined use of nano-magnesium oxide (nano-MgO), an underexplored nanomaterial in geotechnical contexts, and synthetic fibers—a widely recognized mechanical reinforcement method. Through a series of conventional and large-scale laboratory experiments, the effects of various nano-MgO contents and fiber inclusions on the engineering behavior of clay soils were examined. The findings are presented in the following sections using correlative graphical representations and in-depth discussions, with a particular focus on improvements in soil strength, stiffness, and deformation characteristics. In addition, the experimental findings indicate that the combined use of nano-magnesium oxide (NM) and fibers holds potential not only under laboratory conditions but also for field applications. The fine particle size of nano-MgO allows for easy mixing with the soil, while fibers provide physical reinforcement, demonstrating the practicality of this method in situ. In particular, nano-MgO and fiber-amended mixtures prepared at optimum moisture content can be applied directly to low-bearing-capacity clay soils, enhancing both load-bearing capacity and settlement performance. Additionally, the use of low dosages of these additives supports the economic and environmental sustainability of the technique. Therefore, the NM and fiber-based soil improvement approach can be considered a viable and adaptable alternative for field-scale ground improvement across varying soil conditions.

## 2. Materials and Methods

### 2.1. Materials

**Figure 1:**
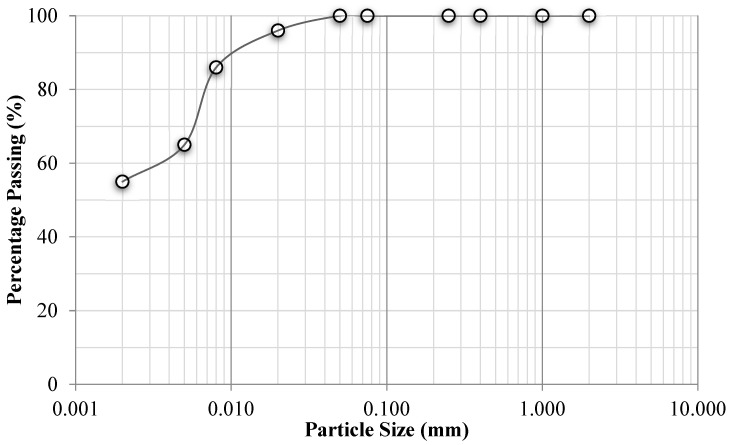
Particle size distribution curve of the clay soil.

The materials utilized in the experiments were clay soil from Adana City, Turkey, and nano-MgO material and fiber material (FBR), both from İstanbul, Turkey. Experiments were performed on the clay samples to assess the engineering properties of the soil in accordance with the relevant ASTM Standards. The particle size distribution curve of the clay soil can be seen in Figure 1 [36].

The specific gravity (27 kN/m^3^), maximum dry unit weight (17.40 kN/m^3^), and optimum water content (18%) of the soil [37] were determined, along with its liquid limit (42%), plastic limit (24%), and plasticity index (18%) [38]. The soil was classified as clay with low plasticity [39]. The following tables demonstrate the results of XRF (Minipal 4) chemical analyses for clay, NM, and FBRs (Table 1, Table 2 and Table 3).

### 2.2. Methods

Block samples were brought from a site in Adana, degraded into smaller sample sizes, and dried in an oven at a constant temperature of 105 ± 5 °C for 24 h. The dried samples were pulverized by a grinding machine. The index properties of the samples taken from the site and prepared at various mixture ratios were identified by traditional laboratory experiments (sieve analysis, wet analysis, pycnometer, and proctor compaction).

Specific ratios of NM were added to the dry clay soil samples and blended until uniform mixtures were obtained. Thereafter, the optimum water content was added in the designated ratios to each sample, and the mixtures were blended again until each sample was uniform.

Additional tests were conducted by adding specified ratios of FBR to the mixtures of NM and clay procured in the same way before adding the water. After the water was added, the prepared sample mixtures were blended until a state of uniformity was attained for each sample. The samples were evenly bagged (5 kg) and kept in a curing room for 24 h to prevent any moisture loss. Finally, the samples were subjected to the tests described below.

**Figure 2:**
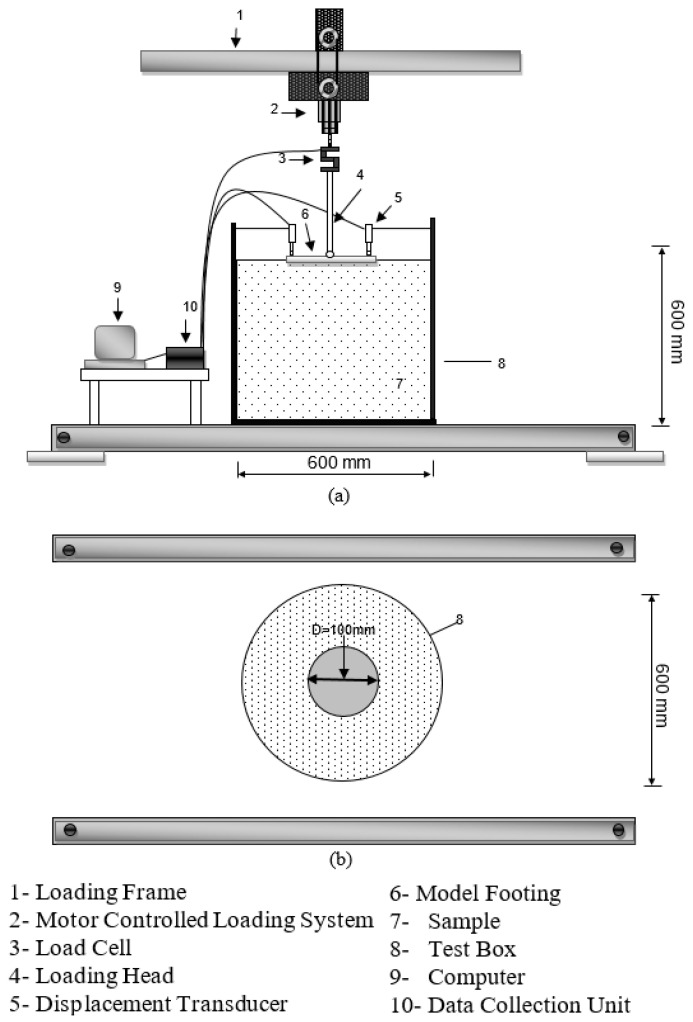
Model testing system: (**a**) plan; (**b**) section.

The cylindrical model test box used was 600 mm high, 600 mm in diameter, and manufactured from 10 mm thick steel profiles (Figure 2). A circular base plate of 100 mm diameter (model footing plate) was placed at the exact center of the test box on top of the sample. Soil samples, each 25 mm thick, were compacted under steady energy by dropping a custom-made 2 kg hammer from a height of 200 mm [42,43] onto the base plate 80 times for each sample layer. The steps required to control water content and surface uniformity were followed for each sample layer.

Two electronic load cells (ESIT Inc. Istanbul, Turkey) were mounted in the test box to record the vertically applied loads and electronic vertical displacement transducers (ELE Inc., Milton Keynes, UK) to read settlements at two points evenly spaced from the center of the model footing. Displacements were taken as the average of the two readings. Before initiating the model tests, reading values were set to zero for the measurement system, and conclusive controlling steps were carried out to ensure the uniformity of the load application and the rest of the system. Loads were exerted vertically and statically on the model footing. The attained load and displacement values were transmitted to a 32-port data collection device and converted to numeric values by DS7 geotechnical software (DataSystem 7.3) in a computer environment. After each test, vertical load, settlement, and stress values were assessed.

For the chosen test box, the dimensions of the model testing case were specified to prevent any boundary effect. Strain meters were installed for that purpose on the edges of the test box before the model footing was loaded. Monitoring that all the stress values read as zero after the application of loads ensured that the boundary effect did not interfere with the accuracy of the testing procedure [5,7].

A comprehensive depiction of the experimental apparatus utilized in this investigation is illustrated in Figure 2. The system was purpose-built to analyze the settlement characteristics of soil specimens under vertical loading conditions, replicating the behavior of shallow foundations. The testing apparatus is mounted on a robust steel framework assembled using bolted connections, which ensures rigidity and inhibits any lateral or torsional shifts during loading procedures. At the upper section of the frame, a servo-driven actuator is positioned to apply gradually increasing vertical loads in a controlled manner. This actuator delivers force via a vertically aligned steel rod, which transfers the load to a stainless-steel footing plate resting directly atop the soil surface. The application of load is managed through displacement control, facilitating precise adjustment and enabling the accurate observation of settlement progression. To monitor the applied force with precision, a strain-gauge-based load cell (model: ESIT LCT series) is installed inline between the actuator and the loading rod. This sensor continuously measures vertical load and transmits data to the acquisition system for live tracking and evaluation. The simulated foundation plate is constructed from stainless steel, measuring 100 mm in width and length, and 20 mm in thickness. Its base is precision-machined to guarantee even load dispersion over the soil contact area. The soil sample beneath the plate is prepared within a cylindrical mold, 600 mm in diameter and height, representing in situ conditions while maintaining strict laboratory control. Vertical deformations are captured using two high-precision Linear Variable Differential Transformers (LVDTs) (model: ELE International. Milton Keynes, UK), positioned symmetrically on opposite sides of the plate. These sensors are affixed to separate stationary frames to eliminate noise from structural vibrations or unintended lateral motion, thereby enhancing measurement reliability. Dual LVDTs also allow the detection of any asymmetrical settlement or tilting during the loading process. All load and displacement data are gathered using a 32-channel data acquisition system, operating at a sampling rate of 10 Hz and within a ±10 V signal range. The system includes modules for signal conditioning, analog-to-digital conversion, and digital data logging, allowing the real-time visualization of load-settlement behavior as well as in-depth post-experiment analysis. The tests were carried out under stable environmental conditions—specifically, at room temperature (20 ± 2 °C) and relative humidity levels of 60–65%—to minimize external influences on soil response. Each specimen was preconditioned for 24 h before testing, and three tests were conducted per soil mix to ensure the consistency and repeatability of the results. Prior to testing, all mechanical elements and sensors were calibrated. Figure 2 presents a detailed schematic of the entire experimental configuration and the interconnection of its components.

## 3. Results

### 3.1. Effects of Water Content and Mixing Ratio

**Figure 3:**
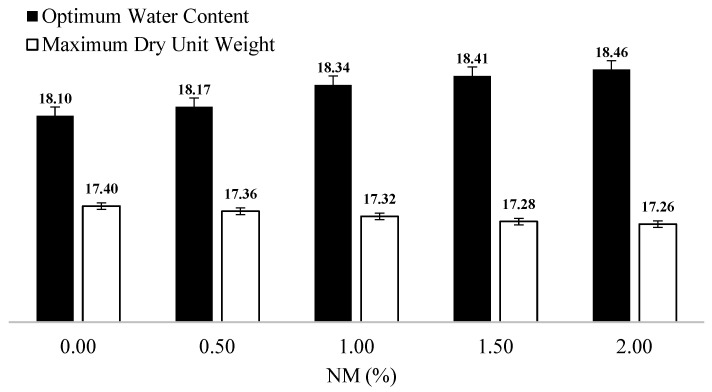
Optimum water content and maximum dry unit weight values for various NM (nano-MgO) ratios.

Compaction tests determined the optimum water content values as 18.10%, 18.17%, 18.34%, 18.41% and 18.46%, respectively, for NM–soil mixtures prepared at ratios of 0.00%, 0.50%, 1.00%, 1.50% and 2.00% (Figure 3). No significant degrees of variations were observed in the unit weight values as the ratio of NM in the mixtures increased. The reason for this phenomenon is thought to be the relatively low ratios of NM in the sample mixtures. On the other hand, the optimum water content values increased as the ratios of NM were increased in the mixtures. It is thought that this is the result of the considerable water absorption capabilities of the Mg and Ca components in NM.

### 3.2. Effect of Fiber (FBR)

**Figure 4:**
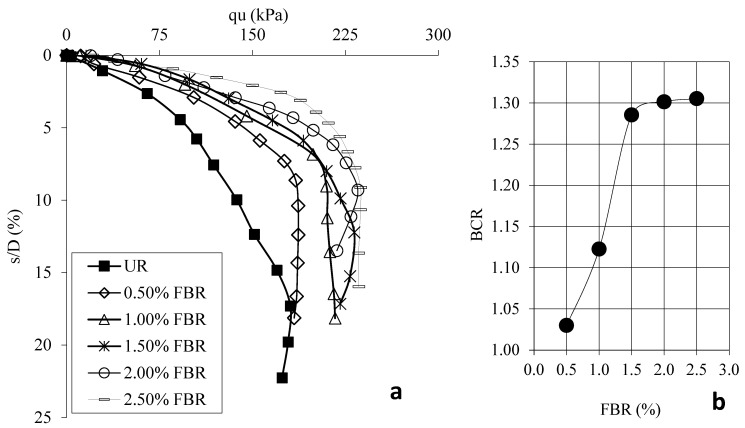
(**a**) q_u_-s/D for various ratios of 30 mm FBRs; (**b**) BCR values for various ratios of 30 mm FBRs (q_u_: applied load, s/D: settlement-to-depth ratio, BCR: bearing capacity ratio, UR: unreinforced clay, FBR: fiber).

**Figure 5:**
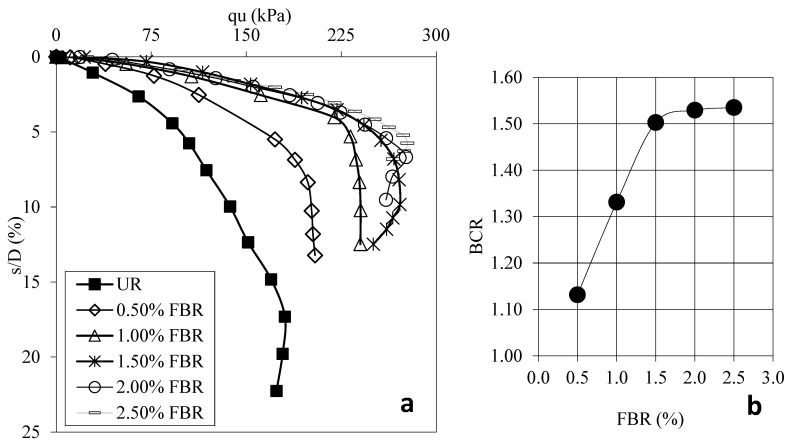
(**a**) q_u_-s/D for various ratios of 60 mm FBRs; (**b**) BCR values for various ratios of 60 mm FBRs (q_u_: applied load, s/D: settlement-to-depth ratio, BCR: bearing capacity ratio, UR: unreinforced clay, FBR: fiber).

**Figure 6:**
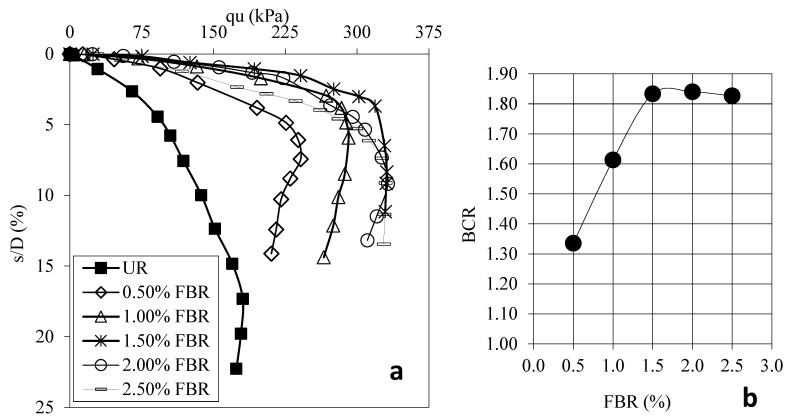
(**a**) q_u_-s/D for various ratios of 90 mm FBRs; (**b**) BCR values for various ratios of 90 mm FBRs (q_u_: applied load, s/D: settlement-to-depth ratio, BCR: bearing capacity ratio, UR: unreinforced clay, FBR: fiber).

**Figure 7:**
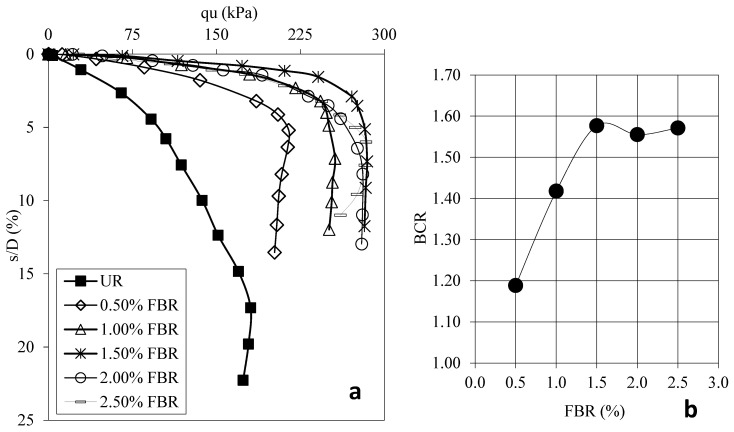
(**a**) q_u_-s/D for various ratios of 120 mm FBRs; (**b**) BCR values for various ratios of 120 mm FBRs (q_u_: applied load, s/D: settlement-to-depth ratio, BCR: bearing capacity ratio, UR: unreinforced clay, FBR: fiber).

Figure 4a shows the load displacement graphs for samples prepared using 30 mm FBR without FBR and different ratios. Figure 4b shows the BCR values corresponding to different FBR ratios. Figure 5a shows the load displacement graphs for samples prepared using 60 mm FBR without FBR and different ratios. Figure 5b shows the BCR values corresponding to different FBR ratios. Figure 6a shows the load displacement graphs for samples prepared using 90 mm FBR without FBR and different ratios. Figure 6b shows the BCR values corresponding to different FBR ratios. Figure 7a shows the load displacement graphs for samples prepared using 120 mm FBR without FBR and different ratios. In Figure 7b, there are BCR values corresponding to different FBR ratios.

Fibers of different lengths (30 mm, 60 mm, 90 mm, and 120 mm) were added to clay soil at ratios of 0.5%, 1.0%, 1.5%, 2.0%, and 2.5%. Adding 30 mm fibers increased the soil’s bearing capacity by about 1.03 to 1.31 times compared to soil without fibers (Figure 4). For 60 mm fibers, the increase was between 1.13 and 1.54 times (Figure 5). With 90 mm fibers, the improvement was even higher, from 1.34 up to 1.84 times (Figure 6). The 120 mm fibers gave an increase between 1.19 and 1.58 times (Figure 7). These results show that adding fibers makes the soil stronger, and longer fibers generally give better improvements.

As the fiber length increased, the soil’s movement under load decreased, meaning the soil became more stable.

When considering both the cost and the strength gained, the best choice is to use 90 mm fibers at a 1.5% ratio. Adding fibers helps the soil resist pressure and pulling forces from the foundation. This is because fibers are flexible and spread out these forces inside the soil, making the structure more durable.

### 3.3. Effect of Nano-MgO (NM)

**Figure 8:**
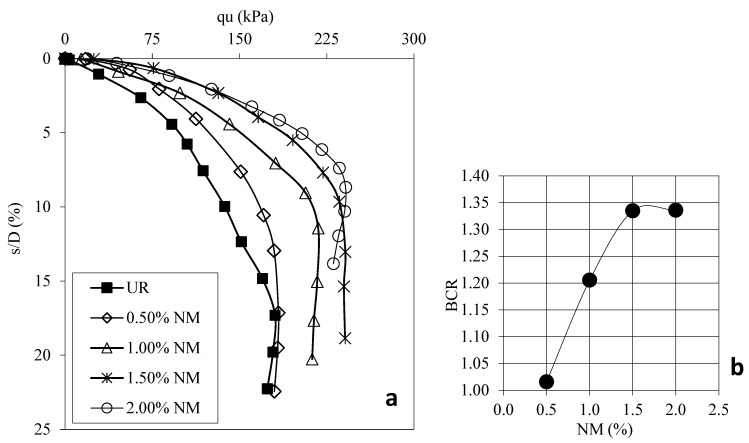
(**a**) q_u_-s/D for various NM ratios; (**b**) BCR values for various NM ratios (q_u_: applied load, s/D: settlement-to-depth ratio, BCR: bearing capacity ratio, UR: unreinforced clay, NM: nano-MgO).

Figure 8a shows the load displacement graphs for samples prepared without NM and using NM at different ratios. Figure 8b shows the BCR values corresponding to different FBR ratios.

The bearing capacity increments were 1.02, 1.21, 1.33, and 1.34 times for NM additions of 0.5%, 1.0%, 1.5%, and 2.0%, respectively (Figure 8). The optimum NM percentage was 1.5%, improving the bearing capacity by 1.33 times. There were no significant improvements for relatively low ratios of NM addition, but the effects increased at higher ratios. However, there were little settlement improvements. Therefore, NM added to plain soil samples improves both bearing capacity and settlement values. Increasing the ratios of NM in the mixtures resulted in more improvements in bearing capacities than in settlement values.

### 3.4. Effect of Nano-MgO (NM) with FBR

**Figure 9:**
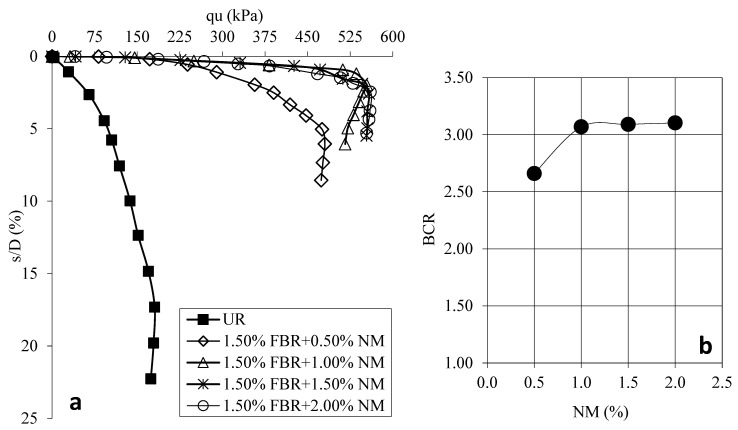
(**a**) q_u_-s/D for various NM ratios at the optimum FBR ratio; (**b**) BCR values for various NM ratios at the optimum FBR ratio (q_u_: applied load, s/D: settlement-to-depth ratio, BCR: bearing capacity ratio, UR: unreinforced clay, NM: nano-MgO).

Figure 9a shows the load displacement graphs for samples prepared using NM at different ratios with FBR. Figure 9b shows the BCR values corresponding to NM ratios at different ratios with FBR.

FBRs at their optimal length and proportion, combined with varying amounts of nanomaterial (NM) at 0.5%, 1.0%, 1.5%, and 2.0%, resulted in a significant increase in bearing capacity—up to 2.66, 3.07, 3.09, and 3.10 times greater than that of untreated plain clay samples, respectively (Figure 9). When balancing both economic feasibility and mechanical performance, the ideal NM content was determined to be 1.0%. In addition to these improvements in bearing capacity, substantial reductions in settlement were also achieved by using the two materials together. The synergistic effect of NM and FBRs yielded the most favorable results, enhancing both the load-bearing capacity and minimizing deformation under load. While the addition of NM predominantly enhanced bearing capacity more significantly than it reduced settlement, the incorporation of FBRs contributed positively to both bearing capacity and settlement mitigation. The NM improved the interfacial bonding between the soil particles and the FBRs, leading to a more cohesive matrix. Meanwhile, the inherent flexibility of FBRs allowed for better distribution and dissipation of tensile and compressive stresses within the soil structure, thereby increasing the durability and stability of the treated soil samples.

### 3.5. Overall Representation for NM and FBR Implementation

**Figure 10:**
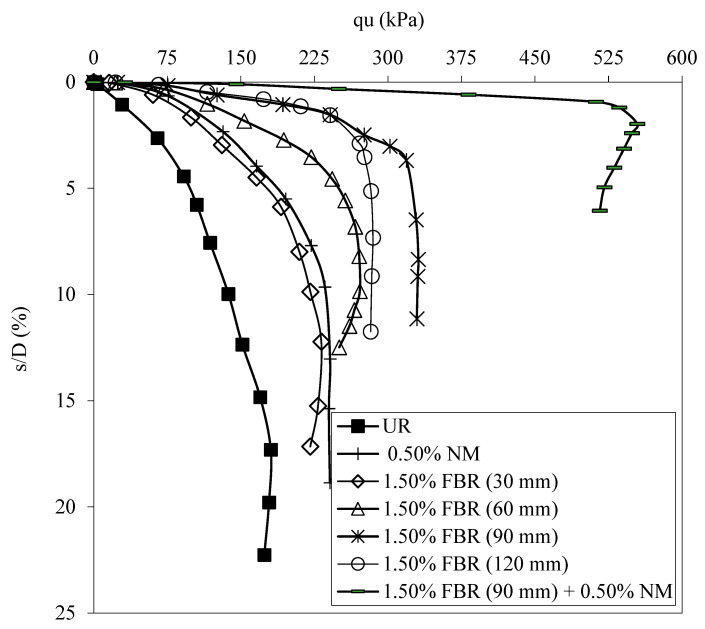
q_u_-s/D values for various NM and FBR ratios (q_u_: applied load, s/D: settlement-to-depth ratio, UR: unreinforced clay, NM: nano-MgO, FBR: fiber).

**Figure 11:**
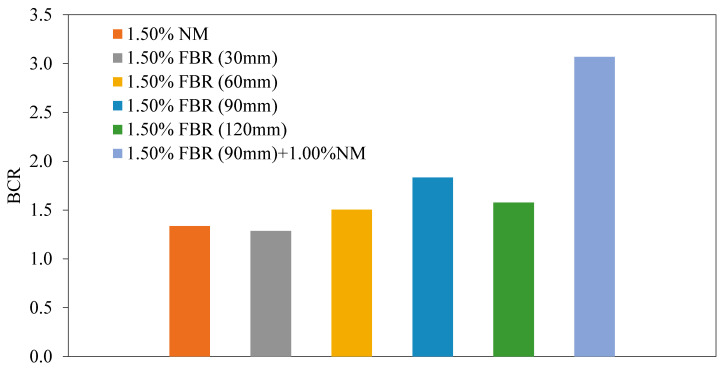
BCR values for various NM and FBR ratios (BCR: bearing capacity ratio, NM: nano-MgO, FBR: fiber).

Figure 10 shows the load displacement graphs for the samples with unreinforced soil, optimum NM ratio, different FBR lengths and ratios, optimum FBR ratio, and optimum NM ratio. Figure 11 shows the BCR values of the mixtures mentioned above.

Figure 10 and Figure 11 present detailed results on the influence of incorporating optimal proportions of fibers and nanomaterials (NM) into clay soil on its bearing capacity. The sole addition of NM at an optimal dosage of 1.5% resulted in a 1.33-fold increase in the soil’s bearing capacity, indicating a notable enhancement in soil strength due to nanomaterial-induced particle bonding and densification. Across all tested fiber lengths—30 mm, 60 mm, 90 mm, and 120 mm—the optimal NM content was consistently 1.5%, yielding bearing capacity ratios (BCRs) of 1.28, 1.50, 1.83, and 1.58, respectively. These results highlight that longer fibers, particularly at 90 mm, contributed more significantly to soil reinforcement, likely due to improved fiber–soil interlocking and tensile load transfer.

When both fibers and NM were combined at their optimum proportions, the bearing capacity improved dramatically—up to 3.07 times that of untreated clay—demonstrating a strong synergistic effect. This enhancement can be attributed to NM’s role in strengthening the soil matrix by promoting better particle bonding and cohesion, while fibers acted as micro-reinforcements that effectively dissipated tensile and compressive stresses through their flexibility and tensile strength. This dual mechanism not only increased the soil’s load-bearing capacity but also improved its deformation characteristics by reducing settlement under load.

Therefore, the integrated application of fibers and NM in clay soils represents an optimal stabilization strategy, achieving superior mechanical performance through combined physicochemical bonding and mechanical reinforcement.

### 3.6. Effect of the Temperature of Mixing Water

**Figure 12:**
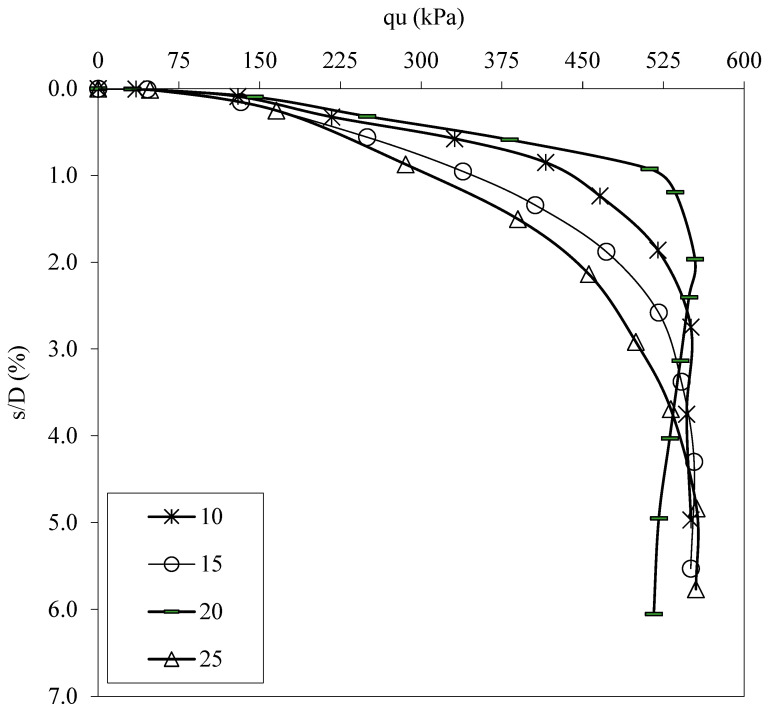
q_u_-s/D values for various temperatures (°C) of mixing water (q_u_: applied load, s/D: settlement-to-depth ratio).

**Figure 13:**
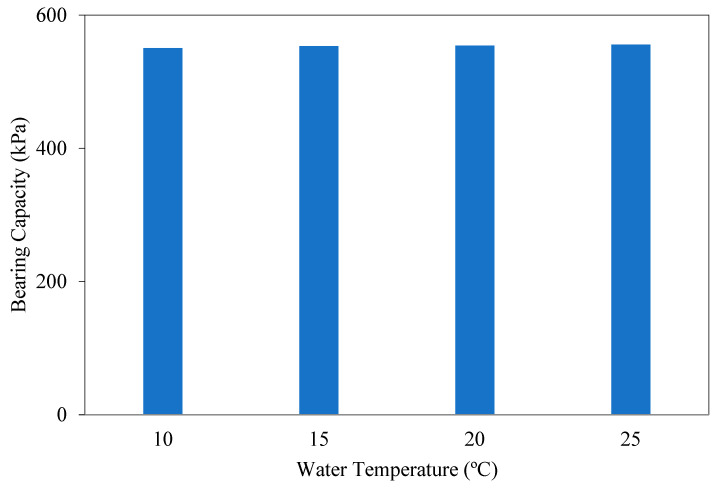
Bearing capacity values for various temperatures of mixing water.

Figure 12 shows the load displacement graphs of soils in optimum FBR and optimum NM mixtures in different temperature environments. Figure 13 shows the BCR values of the mixtures mentioned above.

Sample mixtures were prepared by mixing optimum ratios of FBR and NM to clay under mixing temperatures of 10 °C, 15 °C, 20 °C and 25 °C. The bearing capacity values for water at 15 °C, 20 °C, and 25 °C were 1.0054, 1.0068, and 1.0097 times that of water at 10 °C (Figure 12 and Figure 13). Therefore, the temperature of the mixing water did not have a significant effect on the bearing capacity improvements. It was concluded that the temperature of the water has no significant effect on the reaction taking place during the bonding between NM and FBR.

### 3.7. Effect of Curing Duration

**Figure 14:**
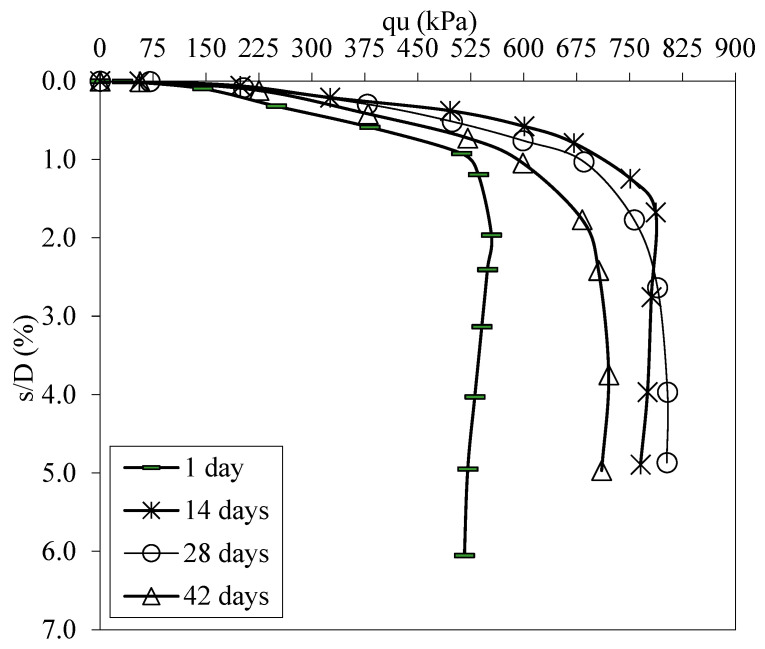
q_u_-s/D values for different periods (q_u_: applied load, s/D: settlement-to-depth ratio).

**Figure 15:**
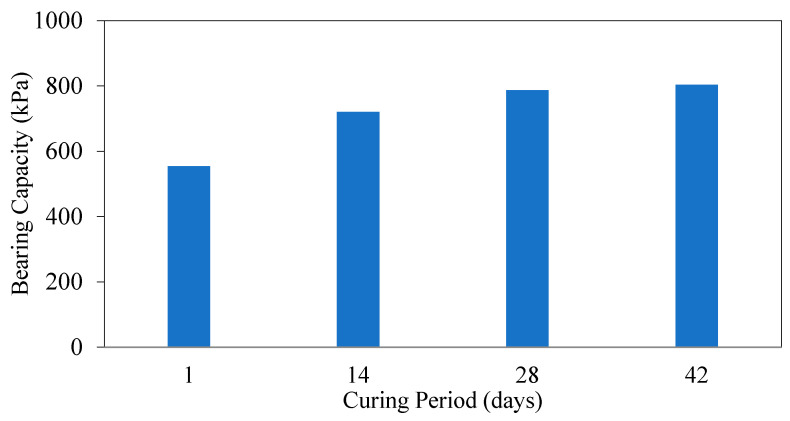
Bearing capacity values for different curing periods.

Figure 14 shows the load displacement graphs of soils in optimum FBR and optimum NM mixtures at different curing times. Figure 15 shows the BCR values of the mixtures mentioned above.

Samples prepared by blending optimal ratios of FBR and NM with clay were subjected to curing periods of 1, 14, 28, and 42 days to evaluate time-dependent changes in mechanical properties. The bearing capacity of samples cured for 14, 28, and 42 days improved by factors of 1.29, 1.41, and 1.44, respectively, relative to the baseline sample cured for only 1 day (Figure 14 and Figure 15). This progressive enhancement demonstrates that extended curing durations significantly influence the development of soil strength. Notably, the rate of bearing capacity improvement was pronounced up to 28 days, after which it plateaued, indicating a diminishing return on strength gains with further curing.

The time-dependent improvement is predominantly attributed to the physicochemical interactions between NM and the clay minerals. Specifically, the nanomaterial facilitates pozzolanic reactions and filler effects, promoting the formation of additional cementitious compounds such as calcium silicate hydrates (CSH) that enhance particle bonding and soil matrix densification. These chemical processes continue to evolve during curing, thereby incrementally increasing the soil’s load-bearing capacity and reducing settlement.

Conversely, FBRs contribute to mechanical reinforcement through immediate physical mechanisms, such as bridging soil particles and distributing tensile and compressive stresses more evenly. Since there is no chemical reaction between FBRs and soil particles, their effect is largely independent of curing time and remains relatively constant throughout the curing period. Therefore, while NM-driven chemical bonding strengthens over time, the fibers provide consistent reinforcement, resulting in a combined improvement in both bearing capacity and settlement behavior that benefits from both time-dependent and immediate mechanisms.

## 4. Conclusions

This study comprehensively examined the enhancement of clay soil mechanical properties through the incorporation of FBRs and NM. The goal was to improve the soil’s load-bearing capacity and reduce settlement, critical factors for geotechnical stability. Key findings and technical insights include the following:

- Optimal Fiber Parameters: The study determined that the optimum fiber length and volumetric content were 90 mm and 1.5%, respectively. At these parameters, the unconfined compressive strength (UCS) of the treated clay increased by approximately 1.83 times compared to untreated soil. These fiber dimensions promote effective load transfer and crack bridging within the soil matrix, while remaining practical for mixing and cost-effective for large-scale applications.
- NM Dosage Effects: When NM was added as the sole additive, the ideal dosage was 1.5% by weight. At this concentration, the bearing capacity improved by up to 1.33 times relative to the untreated samples. The mechanism involves the pozzolanic reaction between NM and clay minerals, forming cementitious compounds such as magnesium silicate hydrate (M-S-H), which enhance soil stiffness and cohesion. However, NM alone did not yield significant reductions in settlement, indicating that strength gains do not fully translate into deformation control.
- Combined Use of NM and FBR: The synergistic effect of combining NM (at an optimized dosage of 1.0%) with FBR (1.5% content, 90 mm length) resulted in a substantial 3.07-fold increase in bearing capacity. The combination leverages both mechanical reinforcement from fibers and chemical stabilization from NM. NM improves the interfacial bonding between fibers and soil particles, enhancing stress transfer and mitigating microcrack propagation under load. This synergy leads to improved resistance against both compressive and tensile stresses, effectively controlling settlement and deformation.
- Influence of Mixing Water Temperature: The experiments showed that varying the temperature of the mixing water had no significant impact on the mechanical properties of the treated soils. This suggests that the hydration and pozzolanic reactions involving NM are relatively insensitive to temperature variations within the tested range, simplifying field conditions where precise temperature control is challenging.
- Effect of Curing Time: Prolonged curing duration positively influenced the development of bearing capacity and settlement reduction in samples with NM, reflecting the ongoing formation of cementitious phases over time. In contrast, fiber reinforcement effects were immediate and stable, showing minimal dependence on curing time. This indicates that while chemical stabilization requires time to mature, mechanical reinforcement provides instant structural benefits.
- In conclusion, this research demonstrates that a combined approach of mechanical reinforcement (FBRs) and chemical stabilization (NM) significantly improves the geotechnical performance of clay soils. The optimal ratios identified ensure both economic feasibility and practical applicability for soil improvement projects.
- This study demonstrated that the combined use of NM and FBR offers promising results not only under laboratory conditions but also for field applications in improving low-bearing-capacity clay soils; the fine particle size of NM enables homogeneous mixing with the soil, while fibers provide physical reinforcement, significantly enhancing both load-bearing capacity and settlement performance. Additionally, the effectiveness of these additives at low dosages supports the economic and environmental sustainability of the method, making it a viable and adaptable alternative for ground improvement across various soil conditions.
- Future research is recommended to focus on long-term performance analyses under different climatic and loading conditions, the modeling of data obtained from field applications, and the evaluation of the method’s effectiveness on other soil types.

## Figures and Tables

**Table 1:** Chemical composition of clay.

Content (%)	MgO	Al_2_O_3_	SiO_2_	P_2_O_5_	K_2_O	CaO	MnO	Fe_2_O_3_	Na_2_O	TiO_2_	LL
**Clay**	6.1	18.4	50.6	0.65	3.10	3.20	3.10	8.70	2.50	1.65	3.15

LL: The ratio of undetermined minerals at very low ratios during chemical analysis.

**Table 2:** Chemical composition of NM [40].

Property	Typical Value
Purity (%)	99.5+
Young’s Modulus (Elastic Modulus)	72–80 GPa
Color	White
Average Particle Size (nm)	85
Bulk Density (kN/m^3^)	0.2
True Density (kN/m^3^)	3.6
Color	White
K	% 0.023
Na	% 0.16
Ca	% 0.096

**Table 3:** Properties of FBRs [41].

Property	Typical Value
Tensile Strength	3.4–3.5 GPa
Young’s Modulus (Elastic Modulus)	72–80 GPa
Density	2.60–2.65 g/cm
Thermal Expansion Coefficient	5.0 × 10^−6^/°C
Melting Point	1250–1450 °C
Electrical Conductivity	Insulator (Dielectric)
Moisture Absorption	<0.1%
Alkali Resistance (for AR glass)	High
Fiber Length	3 mm–25 mm (depends on application)
Filament Diameter	10–15 µm
Component	Approximate Percentage (%)
SiO_2_ (Silicon Dioxide)	52–56
Al_2_O_3_ (Aluminum Oxide)	12–16
CaO (Calcium Oxide)	16–25
B_2_O_3_ (Boron Oxide)	5–10
MgO (Magnesium Oxide)	0–5
SiO_2_ (Silicon Dioxide)	52–56
Al_2_O_3_ (Aluminum Oxide)	12–16

## Data Availability

The data are included in the article.
